# Supplementary material for: Machine Learning Accelerates Crystallization for Structure Determination
Source: Angew Chem Int Ed Engl. 2026 May 4;65(25):e1218503. doi: 10.1002/anie.1218503 (PMC13266958; doi:10.1002/anie.1218503)

checkCIF/PLATON report

Structure factors have been supplied for datablock(s) lcz057

THIS REPORT IS FOR GUIDANCE ONLY. IF USED AS PART OF A REVIEW PROCEDURE FOR PUBLICATION, IT SHOULD NOT REPLACE THE EXPERTISE OF AN EXPERIENCED CRYSTALLOGRAPHIC REFEREE.

No syntax errors found. CIF dictionary Interpreting this report

Datablock: lcz057

Bond precision:	C-C = 0.0156 A		Wavelength=1.54184
Cell:	a=11.8389(3)	b=22.7203(4)	c=12.4354(3)
	alpha=90	beta=90	gamma=90
Temperature:	100 K		

	Calculated	Reported
Volume	3344.92(13)	3344.92(13)
Space group	P n m a	P n m a
Hall group	-P 2ac 2n	-P 2ac 2n
Moiety formula	C15 H3 Ag3 F18 N6, C14 H10 O	C7.5 H1.5 Ag1.5 F9 N3, C7 H5 O0.5
Sum formula	C29 H13 Ag3 F18 N6 O	C14.50 H6.50 Ag1.50 F9 N3 O0.50
Mr	1127.06	563.53
Dx,g cm-3	2.238	2.238
Z	4	8
Mu (mm-1)	15.250	15.250
F000	2160.0	2160.0
F000’	2170.30	
h,k,lmax	15,28,15	14,28,15
Nref	3697	3501
Tmin,Tmax	0.122,0.087	0.238,1.000
Tmin’	0.034	

Correction method= # Reported T Limits: Tmin=0.238 Tmax=1.000
AbsCorr = MULTI-SCAN

Data completeness= 0.947 Theta(max)= 78.827

R(reflections)= 0.0712(3069) wR2(reflections)= 0.2060(3501)

S = 1.148 Npar= 265

The following ALERTS were generated. Each ALERT has the format
test-name_ALERT_alert-type_alert-level.
Click on the hyperlinks for more details of the test.

Alert level A

PLAT971_ALERT_2_A Check Calcd Resid. Dens. 0.99Ang From Ag01 4.03 eA-3

Author Response: This Alert is due to presence of residual density in the presence of heavy metal atom (Ag).

PLAT971_ALERT_2_A Check Calcd Resid. Dens. 1.00Ang From Ag02 3.84 eA-3

Author Response: This Alert is due to presence of residual density in the presence of heavy metal atom (Ag).

PLAT971_ALERT_2_A Check Calcd Resid. Dens. 0.90Ang From Ag01 3.57 eA-3

Author Response: This Alert is due to presence of residual density in the presence of heavy metal atom (Ag).

Alert level C

PLAT041_ALERT_1_C Calc. and Reported SumFormula Strings Differ Please Check
Calc: C29 H13 Ag3 F18 N6 O
Rep.: C14.50 H6.50 Ag1.50 F9 N3 O0.50
PLAT042_ALERT_1_C Calc. and Reported MoietyFormula Strings Differ Please Check
Calc: C15 H3 Ag3 F18 N6, C14 H10 O
Rep.: C7.5 H1.5 Ag1.5 F9 N3, C7 H5 O0.5
PLAT094_ALERT_2_C Ratio of Maximum / Minimum Residual Density .... 2.65 Report
PLAT250_ALERT_2_C Large U3/U1 Ratio for <U(i,j)> Tensor(Resd 2) 2.1 Note
PLAT342_ALERT_3_C Low Bond Precision on C-C Bonds .... 0.01562 Ang.
PLAT906_ALERT_3_C Large K Value in the Analysis of Variance ...... 4.749 Check
PLAT911_ALERT_3_C Missing FCF Refl Between Thmin & STh/L= 0.600 46 Report
12 0 0, 8 6 0, 0 12 0, 4 14 0, 0 24 0, 0 26 0,
12 1 1, 12 0 2, 12 1 2, 0 24 2, 11 0 3, 7 12 3,
0 25 3, 12 0 4, 13 0 4, 13 2 4, 13 3 4, 13 4 4,
12 5 4, 12 6 4, 10 8 4, 9 9 4, 13 0 5, 10 8 5,
10 9 5, 10 10 5, 9 11 5, 10 11 5, 11 11 5, 9 12 5,

```
                  (   16 More Missing: see the .ckf listing file)
PLAT934_ALERT_3_C Number of (Iobs-Icalc)/Sigma(W) > 10 Outliers ..          1 Check
                7  1 11,
PLAT971_ALERT_2_C Check Calcd Resid. Dens.  1.13Ang From Ag01            1.98 eA-3
```

Author Response: This Alert is due to presence of residual density in the presence of heavy metal atom (Ag).

```
PLAT971_ALERT_2_C Check Calcd Resid. Dens.  1.08Ang From Ag02            1.59 eA-3
```

Author Response: This Alert is due to presence of residual density in the presence of heavy metal atom (Ag).

```
PLAT972_ALERT_2_C Check Calcd Resid. Dens.  1.73Ang From Ag01           -1.77 eA-3
PLAT972_ALERT_2_C Check Calcd Resid. Dens.  1.09Ang From C00N           -1.63 eA-3
```

Alert level G

```
PLAT003_ALERT_2_G Number of Uiso or U(i,j)  Restrained non-H-Atoms          8 Report
PLAT045_ALERT_1_G Calculated and Reported Z Differ by a Factor ...      0.500 Check
PLAT083_ALERT_2_G SHELXL Second Parameter in WGHT  Unusually Large      98.06 Why ?
PLAT178_ALERT_4_G The CIF-Embedded .res File Contains SIMU Records          1 Report
PLAT188_ALERT_3_G A Non-default SIMU Restraint Value has been used     0.0100 Report
PLAT242_ALERT_2_G Low    'MainMol' Ueq as Compared to Neighbors of       C00R Check
PLAT242_ALERT_2_G Low    'MainMol' Ueq as Compared to Neighbors of       C00U Check
PLAT242_ALERT_2_G Low    'MainMol' Ueq as Compared to Neighbors of       C00V Check
PLAT434_ALERT_2_G Short Inter HL..HL Contact F007     ..F008     .       2.82 Ang.
                                      1/2-x,1-y,-1/2+z  =      2_564 Check
PLAT720_ALERT_4_G Number of Unusual/Non-Standard Labels .........          39 Note
              Ag01    Ag02    F003    F004    O005    F006    F007    F008
              F009    N00A    F00B    N00C    N00D    F00E    F00F    C00G
              C00H    C00I    C00J    C00K    C00L    H00L    C00M    H00M
              C00N    H00A    H00B    C00O    H00O    C00P    C00Q    H00Q
              C00R    C00S    H00S    C00T    H00T    C00U    C00V
PLAT860_ALERT_3_G Number of Least-Squares Restraints ............          48 Note
PLAT883_ALERT_1_G Absent Datum for _atom_sites_solution_primary ..     Please Do !
PLAT910_ALERT_3_G Missing FCF Reflection(s) Below Theta(Min)[Deg]=       4.05 Note
                0  2  0,
PLAT912_ALERT_4_G Missing # of FCF Reflections Above STh/L=  0.600        147 Note
PLAT933_ALERT_2_G Number of HKL-OMIT Records in Embedded .res File          5 Note
               12  0  0,   3  0 15,  12  0  4,  12  1  1,  11  1  9,
PLAT941_ALERT_3_G Average HKL Measurement Multiplicity ..........         3.6 Low
PLAT969_ALERT_5_G The 'Henn et al.' R-Factor-gap value ..........       6.319 Note
              Predicted wR2: Based on SigI**2  3.26 or SHELX Weight 17.93
PLAT978_ALERT_2_G Number C-C Bonds with Positive Residual Density.          0 Info
```

---

3 **ALERT level A** = Most likely a serious problem - resolve or explain
0 **ALERT level B** = A potentially serious problem, consider carefully
12 **ALERT level C** = Check. Ensure it is not caused by an omission or oversight
18 **ALERT level G** = General information/check it is not something unexpected

4 ALERT type 1 CIF construction/syntax error, inconsistent or missing data

```
 17 ALERT type 2 Indicator that the structure model may be wrong or deficient
  8 ALERT type 3 Indicator that the structure quality may be low
  3 ALERT type 4 Improvement, methodology, query or suggestion
  1 ALERT type 5 Informative message, check
```

It is advisable to attempt to resolve as many as possible of the alerts in all categories. Often the minor alerts point to easily fixed oversights, errors and omissions in your CIF or refinement strategy, so attention to these fine details can be worthwhile. It is up to the individual to critically assess their own results and, if necessary, seek expert advice.

Validation response form

Please find below a validation response form (VRF) that can be filled in and pasted into your CIF.

```
# start Validation Reply Form
_vrf_PLAT041_lcz057
;
PROBLEM: Calc. and Reported SumFormula    Strings  Differ     Please Check
RESPONSE: ...
;
_vrf_PLAT042_lcz057
;
PROBLEM: Calc. and Reported MoietyFormula Strings  Differ     Please Check
RESPONSE: ...
;
_vrf_PLAT094_lcz057
;
PROBLEM: Ratio of Maximum / Minimum Residual Density ....       2.65 Report
RESPONSE: ...
;
_vrf_PLAT250_lcz057
;
PROBLEM: Large U3/U1 Ratio for <U(i,j)> Tensor(Resd    2)        2.1 Note
RESPONSE: ...
;
_vrf_PLAT342_lcz057
;
PROBLEM: Low Bond Precision on  C-C Bonds ...............    0.01562 Ang.
RESPONSE: ...
;
_vrf_PLAT906_lcz057
;
PROBLEM: Large K Value in the Analysis of Variance ......      4.749 Check
RESPONSE: ...
;
_vrf_PLAT911_lcz057
;
PROBLEM: Missing FCF Refl Between Thmin & STh/L=    0.600         46 Report
RESPONSE: ...
;
_vrf_PLAT934_lcz057
;
PROBLEM: Number of (Iobs-Icalc)/Sigma(W) > 10 Outliers ..          1 Check
```

```
RESPONSE: ...
;
_vrf_PLAT972_lcz057
;
PROBLEM: Check Calcd Resid. Dens.  1.73Ang From Ag01          -1.77 eA-3
RESPONSE: ...
;
# end Validation Reply Form
```

PLATON version of 17/09/2025; check.def file version of 17/09/2025

---

duplicate check

No duplication found

Datablock lcz057 - ellipsoid plot

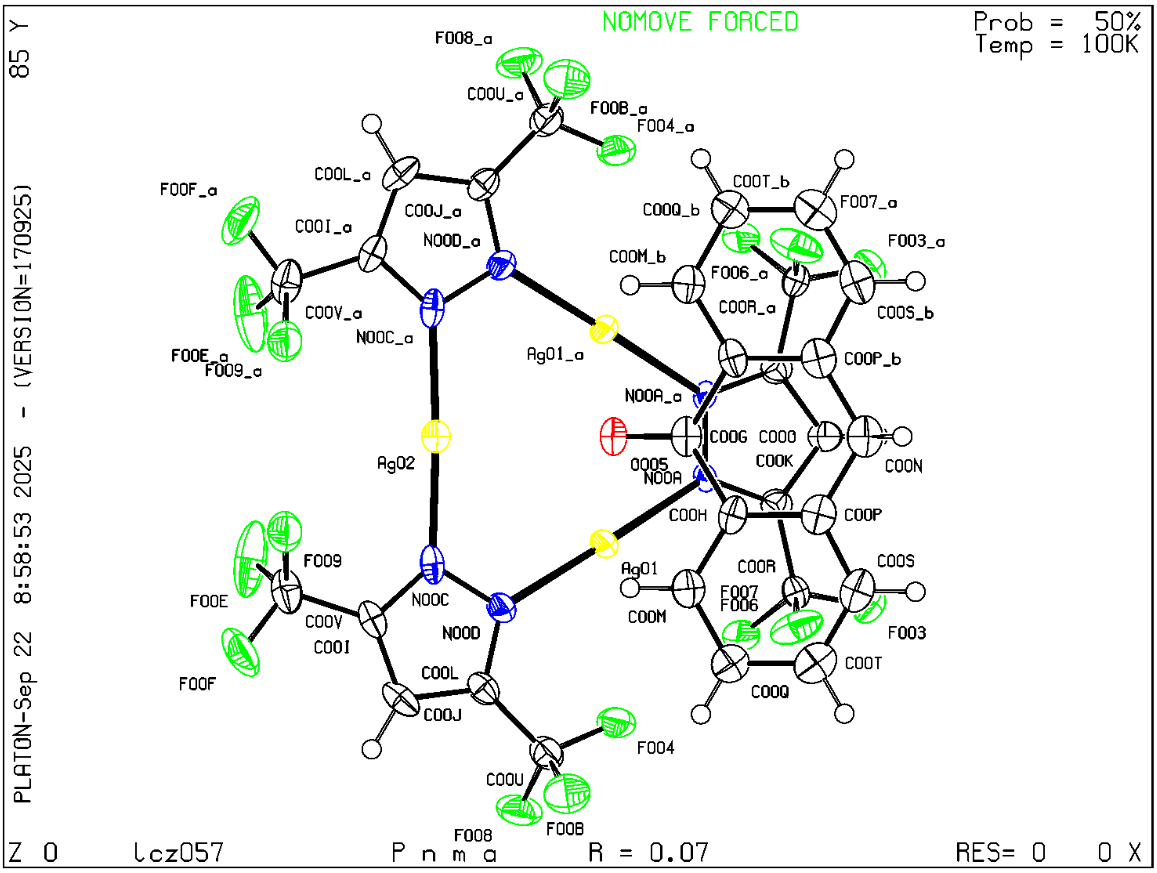

Supplement: Supplementary file 2 — Supporting File 2: anie72462‐sup‐0002‐DataFile.zip. [file ANIE-65-e1218503-s002.zip › supplementary_materials_crystallographic_data submit.zip!/cif_and_checkcif(1-114)/Ag3Pz3_57/Ag3Pz3_57_checkcif.pdf]
